# A New Side Effect of Intravitreal Dexamethasone Implant (Ozurdex®)

**DOI:** 10.1155/2017/6369085

**Published:** 2017-10-02

**Authors:** Erdem Eris, Gurkan Erdogan, Irfan Perente, Gokhan Demir, Burcin Kepez Yildiz, Ebru Demet Aygit

**Affiliations:** Dr. Resat Belger Beyoglu Eye Training and Research Hospital, Istanbul, Turkey

## Abstract

Dexamethasone implant, 0,7 mg (Ozurdex, Allergan, Inc., Irvine, CA, USA), is drug mostly used in the treatment of the diabetic macular edema and edema related to retinal vein occlusion. By reporting this case we aimed to report a new side effect of 0.7 mg intravitreal dexamethasone implant that has not been reported in the literature before.

## 1. Introduction

Central serous chorioretinopathy (CSC) is a disorder characterized by serous retinal detachment and/or retinal pigment epithelial (RPE) detachment, changes that are most often confined to the macula and due to leakage of fluid through the RPE into the subretinal space [1, 2]. CSC is a relatively rare cause of visual impairment, which typically affects the adult males (20–50 years) [3]. No case has been reported in individuals younger than twenty before [4]. CSC has been associated with the use of agents such as endogenous hypercortisolism and epinephrine. Iatrogenically systemic corticosteroids have been charged as a cause of CSC [5]. But CSC after local intraocular dexamethasone implantation has not ever been reported yet. The overactivation of mineralocorticoid receptor pathway in choroid vessels is still unknown etiology for CSC [6]. Hypertension, pregnancy, dialysis, organ transplantation, and systemic lupus erythematosus are entities that could result in choroidal vascular dysfunction and CSC [5, 6].

To the best of our knowledge, this is the first case report in the literature where CSC occurred following intraocular dexamethasone implantation.

## 2. Case Report

**Figure 1:**
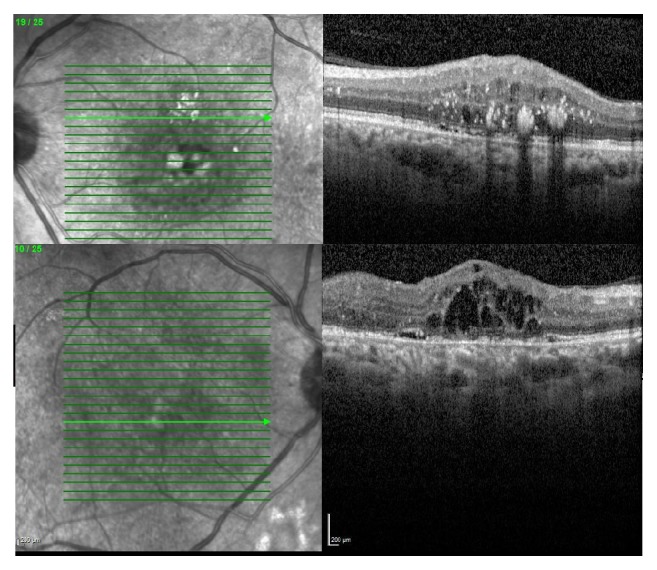
Macular edema secondary to diabetic retinopathy was seen on left eye's OCT image (superior image). Macular edema secondary to diabetic retinopathy was seen on right eye's OCT image (inferior image).

**Figure 2:**
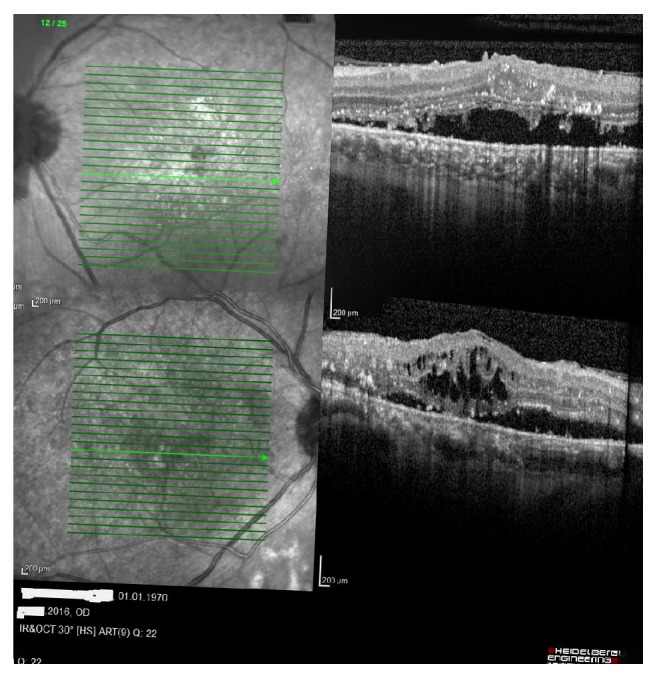
Central serous chorioretinopathy was seen on left eye's OCT image (superior image). Central serous chorioretinopathy was seen on right eye's OCT image (inferior image).

**Figure 3:**
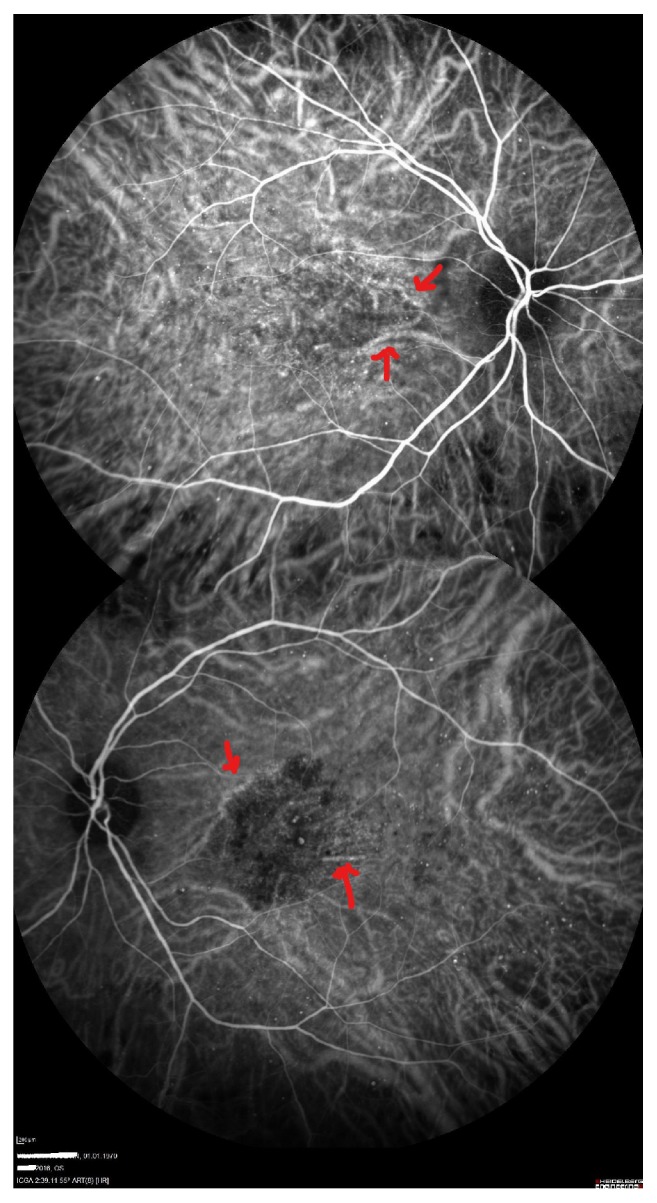
Indocyanine Green Angiography images (superior image is right eye and inferior image is left eye). Red arrows show choroidal vascular enlargement.

**Figure 4:**
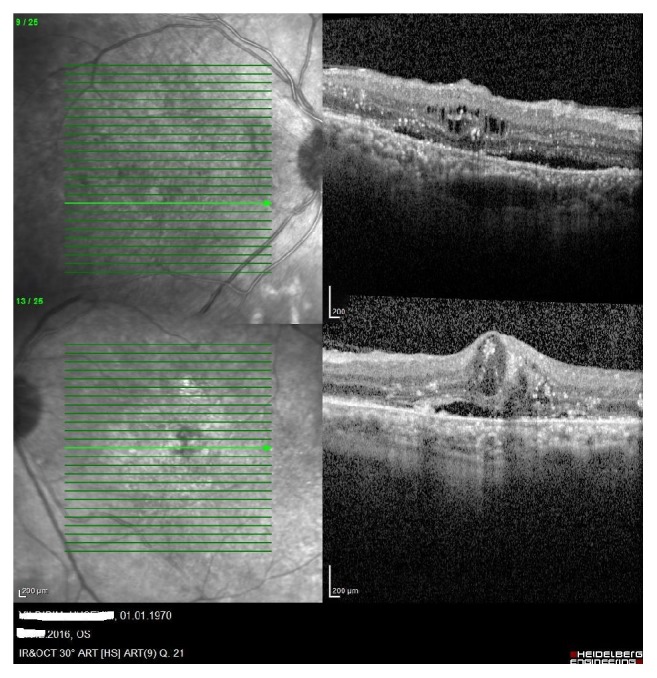
Serous retinal detachments levels were decreased (superior image is right eye and inferior image is left eye).

**Figure 5:**
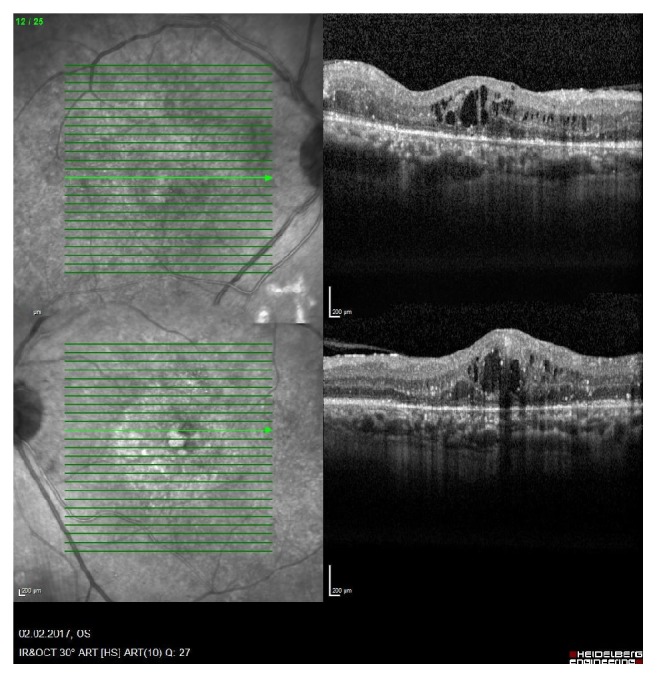
Three months later both eye's serous retinal detachments disappeared (superior image is right eye and inferior image is left eye).

A 46-year-old male patient with a history of diabetes mellitus applied to our clinic with best corrected visual acuity (BCVA) of 80/200 for the right eye (OD) and 40/200 for the left one (OS) (Figure 1). The patient had applied to both eyes the 0.7 mg dexamethasone intravitreal implant (Ozurdex) for the treatment of diabetic macular edema. After a month of these applications, the best corrected visual acuities were 40/200 OD and 10/200 OS. Fundus examination revealed a macular serous detachment of both eyes. Both eyes had a large serous neurosensory detachment on optical coherence tomography (OCT) (Heidelberg Engineering, Heidelberg, Germany) (Figure 2). Although typical CSC findings (round, well-delineated serous detachment, massive choroid thickness, and pigment epithelial detachment [PED]) were not seen on OCT, other differential diagnoses of CSC were eliminated by fundus fluorescein angiography (FFA), Indocyanine Green Angiography (ICGA), and fundus examination. On ICGA there was only choroidal vascular enlargement (Figure 3). Upon slit lamp examination, there was no evidence of inflammation in the anterior or posterior chamber. There were no notable findings that can cause CSC performed in the Endocrine Department. The patient was diagnosed with dexamethasone intravitreal implant induced CSC, whose clinical course was monitored without any specific treatments. Two months following a monitoring regimen, fundoscopy and OCT were performed (Heidelberg Engineering, Heidelberg, Germany). Serous retinal detachments levels had decreased and the best corrected visual acuity in the both eyes had increased (Figure 4). Three months later both eye's serous retinal detachments had disappeared (Figure 5). Final BCVA were 100/200 OD and 50/200 OS.

## 3. Discussion

Central serous chorioretinopathy is a chorioretinal disease whose pathogenesis remains obscure; however recent studies show its vascular origin. The reduced choroidal perfusion is believed to occur secondary to vasomotor instability or sympathetic nervous system excitation [4, 5].

CSC mostly occurs between 20 and 45 years of age. That is because if patients are over 50 years then macular degeneration, choroidal neovascularization, or polypoidal choroid vasculopathy should be ruled out for CSC diagnosis [7]. OCT and ICG may help to differentiate from other diagnoses [8]. In our case, other differential diagnoses of CSC were eliminated with fundus fluorescein angiography (FFA), Indocyanine Green Angiography (ICGA), and fundus examination.

But approximately 50% of patients may develop recurrence [9] and a chronic disease that can produce decreased visual acuity. Chronic CSC could be treated by the effectiveness of laser therapy and photodynamic therapy (PDT). Although systemic and topical steroid-induced central serous chorioretinopathy was reported previously, dexamethasone intravitreal implant induced CSC was not reported before [10]. On the other hand two months later although dexamethasone intravitreal implant effect is expected to continue, we observed a decrease on the serous detachment of the CSC. It would be relevant to the peak time of dexamethasone release. But more studies are necessary to cast light on its effect.
